# Supplementary material for: Hot spot prediction in protein-protein interactions by an ensemble system
Source: BMC Syst Biol. 2018 Dec 31;12(Suppl 9):132. doi: 10.1186/s12918-018-0665-8 (PMC6311905; doi:10.1186/s12918-018-0665-8)
Supplement: Supplementary file 2 — All relevant MATLAB code. A simple Matlab implement of our predictor. (ZIP 19,742 kb) [file 12918_2018_665_MOESM2_ESM.zip › Additional file 2/Read ASA/ASA website/SKEMPI/1DANT.htm]

 NetSurfP - Protein Surface Accessibility and Secondary Structure Predictions

  

|  |  |  |
| --- | --- | --- |
|  |  | NetSurfP - Protein Surface Accessibility and Secondary Structure Predictions  Technical University of Denmark |

  


---

```
# For publication of results, please cite:
# A generic method for assignment of reliability scores applied to solvent accessibility predictions.
# Bent Petersen, Thomas Nordahl Petersen, Pernille Andersen, Morten Nielsen and Claus Lundegaard
# BMC Structural Biology 2009, 9:51 doi:10.1186/1472-6807-9-51
#
# Column 1: Class assignment - B for buried or E for Exposed - Threshold: 25% exposure, but not based on RSA
# Column 2: Amino acid
# Column 3: Sequence name
# Column 4: Amino acid number
# Column 5: Relative Surface Accessibility - RSA
# Column 6: Absolute Surface Accessibility
# Column 7: Z-fit score for RSA prediction
# Column 8: Probability for Alpha-Helix
# Column 9: Probability for Beta-strand
# Column 10: Probability for Coil
E T  1DANT                  1    0.848 117.548   0.697   0.003   0.003   0.994
E V  1DANT                  2    0.375  57.607  -1.065   0.020   0.205   0.775
E A  1DANT                  3    0.531  58.494   0.801   0.021   0.451   0.528
B A  1DANT                  4    0.241  26.525  -1.564   0.004   0.616   0.381
E Y  1DANT                  5    0.309  65.969  -0.067   0.003   0.718   0.279
E N  1DANT                  6    0.444  65.060  -0.519   0.003   0.718   0.279
B L  1DANT                  7    0.117  21.404   0.486   0.018   0.846   0.136
E T  1DANT                  8    0.317  43.968   0.324   0.018   0.846   0.136
B W  1DANT                  9    0.126  30.231  -0.088   0.001   0.900   0.099
E K  1DANT                 10    0.384  79.051   0.511   0.001   0.900   0.099
B S  1DANT                 11    0.190  22.303  -1.470   0.023   0.655   0.322
E T  1DANT                 12    0.354  49.030  -0.587   0.005   0.336   0.660
E N  1DANT                 13    0.540  79.115  -0.206   0.004   0.138   0.858
B F  1DANT                 14    0.163  32.694  -0.392   0.020   0.205   0.775
E K  1DANT                 15    0.414  85.160   0.575   0.004   0.616   0.381
B T  1DANT                 16    0.199  27.643   0.378   0.002   0.816   0.182
B I  1DANT                 17    0.107  19.850   0.487   0.011   0.918   0.071
B L  1DANT                 18    0.106  19.409   0.616   0.001   0.900   0.099
E E  1DANT                 19    0.377  65.844   0.071   0.001   0.900   0.099
B W  1DANT                 20    0.106  25.373  -0.133   0.002   0.816   0.182
E E  1DANT                 21    0.432  75.470   0.216   0.004   0.514   0.481
E P  1DANT                 22    0.333  47.196  -0.922   0.005   0.336   0.660
E K  1DANT                 23    0.541 111.243   0.425   0.005   0.336   0.660
E P  1DANT                 24    0.299  42.471  -1.278   0.004   0.138   0.858
E V  1DANT                 25    0.394  60.527  -0.781   0.004   0.085   0.910
E N  1DANT                 26    0.644  94.223  -1.257   0.004   0.085   0.910
B Q  1DANT                 27    0.237  42.364   0.515   0.004   0.420   0.576
E V  1DANT                 28    0.274  42.145  -0.248   0.002   0.816   0.182
B Y  1DANT                 29    0.118  25.238   0.938   0.001   0.900   0.099
B T  1DANT                 30    0.142  19.709   0.642   0.001   0.959   0.040
B V  1DANT                 31    0.046   7.132   1.086   0.001   0.959   0.040
B Q  1DANT                 32    0.272  48.615   0.626   0.001   0.959   0.040
B I  1DANT                 33    0.080  14.819   0.309   0.001   0.900   0.099
B S  1DANT                 34    0.113  13.244  -0.580   0.023   0.655   0.322
E T  1DANT                 35    0.478  66.271  -0.094   0.021   0.451   0.528
E K  1DANT                 36    0.508 104.496   0.144   0.056   0.142   0.802
E S  1DANT                 37    0.487  57.041  -1.806   0.053   0.043   0.903
E G  1DANT                 38    0.659  51.895   0.089   0.053   0.043   0.903
E D  1DANT                 39    0.558  80.365   0.036   0.052   0.084   0.864
B W  1DANT                 40    0.241  57.864   0.586   0.125   0.227   0.648
E K  1DANT                 41    0.528 108.692   0.663   0.135   0.317   0.548
E S  1DANT                 42    0.424  49.705   0.543   0.125   0.227   0.648
E K  1DANT                 43    0.298  61.381   1.373   0.135   0.317   0.548
B C  1DANT                 44    0.058   8.157   0.037   0.079   0.592   0.329
B F  1DANT                 45    0.153  30.687  -0.095   0.085   0.775   0.140
E Y  1DANT                 46    0.342  73.021   0.313   0.087   0.683   0.230
B T  1DANT                 47    0.150  20.791  -0.180   0.074   0.484   0.442
E T  1DANT                 48    0.397  55.008  -0.539   0.064   0.216   0.721
E D  1DANT                 49    0.560  80.754  -0.954   0.113   0.087   0.800
B T  1DANT                 50    0.279  38.683  -0.423   0.191   0.086   0.723
E E  1DANT                 51    0.427  74.544  -0.345   0.307   0.165   0.527
B C  1DANT                 52    0.083  11.583   0.022   0.191   0.086   0.723
E D  1DANT                 53    0.416  59.902   0.089   0.278   0.093   0.628
B L  1DANT                 54    0.137  25.085   0.292   0.354   0.048   0.598
B T  1DANT                 55    0.254  35.188   0.056   0.622   0.015   0.363
E D  1DANT                 56    0.527  75.897  -0.507   0.879   0.010   0.111
E E  1DANT                 57    0.528  92.311   0.497   0.879   0.010   0.111
B I  1DANT                 58    0.079  14.559  -0.484   0.879   0.010   0.111
B V  1DANT                 59    0.235  36.181   0.581   0.802   0.014   0.185
E K  1DANT                 60    0.613 126.032   1.332   0.717   0.014   0.269
E D  1DANT                 61    0.414  59.600   0.201   0.694   0.003   0.303
B V  1DANT                 62    0.059   9.007   0.334   0.622   0.015   0.363
E K  1DANT                 63    0.438  90.035   0.746   0.717   0.014   0.269
E Q  1DANT                 64    0.531  94.819   0.241   0.751   0.050   0.199
E T  1DANT                 65    0.286  39.724   0.466   0.701   0.107   0.192
B Y  1DANT                 66    0.103  22.097   0.944   0.478   0.309   0.214
E L  1DANT                 67    0.286  52.367  -0.134   0.391   0.466   0.143
B A  1DANT                 68    0.145  15.946  -0.221   0.268   0.505   0.227
E R  1DANT                 69    0.347  79.486   0.377   0.268   0.505   0.227
B V  1DANT                 70    0.126  19.366   0.311   0.268   0.505   0.227
B F  1DANT                 71    0.259  51.901  -0.266   0.148   0.418   0.435
E S  1DANT                 72    0.440  51.533  -1.040   0.069   0.386   0.545
E Y  1DANT                 73    0.423  90.395   0.125   0.056   0.142   0.802
E P  1DANT                 74    0.488  69.176  -1.205   0.053   0.043   0.903
E A  1DANT                 75    0.899  99.015  -1.099   0.003   0.003   0.994

Explain the output.  Go back.
```

---
